# Using the Emission of Muonic X-rays as a Spectroscopic Tool for the Investigation of the Local Chemistry of Elements

**DOI:** 10.3390/nano10071260

**Published:** 2020-06-28

**Authors:** Matteo Aramini, Chiara Milanese, Adrian D. Hillier, Alessandro Girella, Christian Horstmann, Thomas Klassen, Katsuo Ishida, Martin Dornheim, Claudio Pistidda

**Affiliations:** 1 UKRI Rutherford Appleton Laboratory, ISIS Pulsed Neutron and Muon Facility, Didcot OX11 0QX, UK; matteo.aramini@diamond.ac.uk (M.A.); adrian.hillier@stfc.ac.uk (A.D.H.); ishida@riken.jp (K.I.); 2 Pavia Hydrogen Lab, Chemistry Department, Physical Chemistry Section, C.S.G.I. and Pavia University, Viale Taramelli, 16, 27100 Pavia, Italy; alessandro.girella@unipv.it; 3 Institute of Materials Research, Materials Technology, Helmholtz-Zentrum Geesthacht GmbH, Max-Planck-Straße 1, 21502 Geesthacht, Germany; christian.horstmann@hzg.de (C.H.); klassen@hsu-hh.de (T.K.); martin.dornheim@hzg.de (M.D.); 4 Institute of Materials Technology, Helmut Schmidt University, Holstenhofweg 85, 22043 Hamburg, Germany; 5 RIKEN Nishina Center, RIKEN, Nishina Bldg., 2-1 Hirosawa, Wako, Saitama 351-0198, Japan

**Keywords:** muonic X-ray emission spectroscopy, hydrogen storage, calcium borohydride, Mg-based materials, synchrotron radiation

## Abstract

There are several techniques providing quantitative elemental analysis, but very few capable of identifying both the concentration and chemical state of elements. This study presents a systematic investigation of the properties of the X-rays emitted after the atomic capture of negatively charged muons. The probability rates of the muonic transitions possess sensitivity to the electronic structure of materials, thus making the muonic X-ray Emission Spectroscopy complementary to the X-ray Absorption and Emission techniques for the study of the chemistry of elements, and able of unparalleled analysis in case of elements bearing low atomic numbers. This qualitative method is applied to the characterization of light elements-based, energy-relevant materials involved in the reaction of hydrogen desorption from the reactive hydride composite Ca(BH_4_)_2_-Mg_2_NiH_4_. The origin of the influence of the band-structure on the muonic atom is discussed and the observed effects are attributed to the contribution of the electronic structure to the screening and to the momentum distribution in the muon cascade.

## 1. Introduction

The chemical identification of materials nowadays largely exploits X-ray techniques. X-ray diffraction (XRD) is the main reference for the structural investigation of compounds exhibiting long-range order [1].

X-ray Absorption Spectroscopy (XAS) provides the possibility to use the local nature of its probe to investigate the oxidation state and the first coordination spheres of a variety of elements [2]. In the footsteps of XAS, X-ray Emission Spectroscopy (XES) has emerged as an important spectroscopic tool, over the past years, with applications focused on the chemistry of catalysis [3], biology [4], and condensed matter [5].

X-ray scattering techniques are best suited for high Z materials, while X-ray scientists have been developing advanced methods to access the elements of the first, second, and third rows of the periodic table [6], such as the use of soft X-ray [7], or more exotic techniques such as X-ray Raman Scattering [8].

The technique of muonic X-ray Emission Spectroscopy (µ-XES) has strong similarities with X-ray Fluorescence (XRF), but presents several advantages that determine its complementary to established X-ray methods. Muon momentum can be changed, in the ISIS facility, within the range from 20 MeV/c to 90 MeV/c, leading to implantation depth from a few µm up to several centimeters and a depth distribution localized within a volume covering 35% (FWHM) of the total depth. These versatile conditions allow for the selection of the region where X-rays are generated [9].

The energies of muonic X-rays are higher than those of X-rays from electronic transitions because of the larger mass of a negative muon (c.a. 207 times larger than the one of electrons), and the energy of the emitted radiation falls in the range from 2 keV for hydrogen up to approximately 6000 keV for lead, when considering the K lines only [10].

Hence, these hard X-rays can travel through condensed matter without significant photon self-absorption, making the measurement of light elements possible even in the case of in-situ reactive environments and sealed sample holders. The consequence is that virtually all the elements of the periodic table with Z starting from lithium are quantitatively accessible with the same technique [11].

Despite the similarities in the techniques, can µ-XES access the same range of chemical information of the more established X-ray Emission spectroscopy?

When a µ- stops in matter, it is captured in the Coulomb field of a nucleus and a muonic atom is formed [11]. In the last century, several muon-capture models were developed in order to explain the complex behavior of atomic capture rate and its nuclear and isotopic dependence beyond Fermi-Teller approximation [12,13,14].

**Figure 1:**
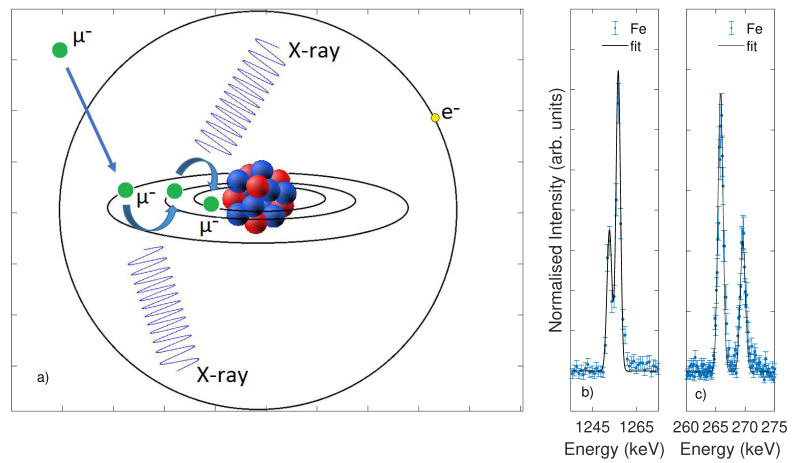
Schematic representation of the muonic atom, the cascade process, and the consequent emission of X-rays (**a**). Selected muonic X-ray spectra of iron compounds at the Kα**_1_** and Kα**_2_** emission lines (**b**) and at the Lα**_1_**-Lα**_2_** and Lβ**_1_** emission lines (**c**).

The captured muon rapidly de-excites to the muonic 1s state through emission of Auger electron and characteristic muonic X-ray. Extensive efforts have been devoted to the understanding of the cascade process, leading to formulation of a general µ- cascade model [15,16,17,18]. The nuclear structure determines different muonic energy levels, and the muonic X-rays corresponding to the transitions among these levels can be easily identified and unambiguously attributed to the elements. Hereafter, Figure 1 shows a schematic representation of the cascade process and consequent emission of X-rays.

The technique was thus immediately exploited as a method for elemental analysis [19], and proofs of concept have been recently provided by the studies of position- and depth-dependent elemental composition in ancient coins, bronze, and asteroids [10,20,21,22]. However, the fine details of the formation of the muonic atom, such as the atomic and molecular effects of the capture process, have not been investigated. Previous studies hinted at the possibility of the chemical sensitivity of muonic X-rays. Ninomiya and coauthors reported changes in the Kα and Kβ intensities in the muonic X-rays from gas under different gas densities [23]. Knight and coauthors showed a similar feature in carbon-based solid-state materials [24]. Nevertheless, early investigations focused solely on the details of the capture ratio among elements and the influence of ionic radius and charge on the emitted lines [25]. The application of the technique has been mostly focused on the elemental analysis, and its analogy with XES in terms of chemical sensitivity has been hinted at in previous research but never thoroughly investigated. This pioneering work shows, for the first time (to our knowledge), the sensitivity of µ-XES to the electronic-structure and the possibility to exploit such a feature as a fingerprint method for the chemical identification of compounds in complex chemical reactions. Two independent sets of data are presented: the former introduces the results collected on oxides of a transition metal (Fe), while the latter presents µ-XES results from light elements (B, Ca) of interest for energy-storage materials. The results are explained by discussing the interplay between µ-XES and the local electronic-structure with respect to the physics of the generation of emission lines.

## 2. Materials and Methods

The solid-state materials used in this work as standards and/or as reactants were purchased in powder form, with the highest available degree of purity, from commercially available suppliers. CaH_2_ (98%), NaBH_4_ (98%), MgB_2_ (95%), Ni (99.8%), MgH_2_ (98%), and CaB_6_ (99.5%) were purchased from Alfa Aesar. Ca(BH_4_)_2_ (95%), amorphous B (95%), crystalline B (99.9%), LiBH_4_ (95%), Ca (99%), FeO (99,7%), FeBr_3_ (98%), FeCl_3_ (99.9%), and FeF_3_ (99%) were purchased from Sigma-Aldrich (St. Louis, Missouri, United States). CaB_12_H_12_ (95%) was purchased from KatChem (Prague, Czech Republic). D_2_ (99.9%) was purchased from Linde (Dublin, Ireland). Mg_2_NiH_4_, MgNi_2.5_B_2_ and CaD_2_ are not commercially available and were synthesized in house. All materials were stored and handled in dedicated argon-filled gloveboxes under a continuously purified argon flow (O_2_ and H_2_O levels < 1 ppm). Mg_2_NiH_4_ [26,27] was synthesized from a mixture of MgH_2_ and Ni in the molar ratio of 2:1. The mixture was milled in an Al_2_O_3_ vial for 4 h using a FRITSCH P6 planetary mill (Idar-Oberstein, Germany), a ball to powder ratio of 5:1, and a rotation speed of 300 rpm. After milling, the mixture was transferred in a stainless steel autoclave from Parr Instrument Company (Moline, Illinois, United States) and heated up to 400 °C under a final hydrogen pressure of 225 bar. The material was kept under these temperatures and hydrogen pressure conditions for 24 h. The precautionary application of a hydrogen back pressure to the synthesis of Mg_2_NiH_4_ was aimed at preventing any possible phase decomposition upon heating. MgNi_2.5_B_2_ [28,29] was prepared starting from a mixture of Ni and MgB_2_ in the ratio 2,5:1. The mixture was milled for 60 min in a stainless steel vial using a SPEX 8000M Mixer/Mill (Metuchen, New Jersey, United States) and a ball to powder ratio of 10:1. The milled material was then annealed at 850 °C in argon atmosphere for 24 h. In order to prepare CaD_2_, 2 g of metallic Ca was charged in a stainless steel high pressure vial from Evico Magnetic (Dresden, Germany) and then milled for 24 h under an initial D_2_ pressure of 40 bar using a FRITSCH P6 planetary mill, a ball to powder ratio of 10:1, and a rotation speed of 450 rpm. For the material labelled as “Sample”, a 2 g mixture of Ca(BH_4_)_2_ and Mg_2_NiH_4_ in the ratio 1:2.5 was milled for 60 min in a stainless steel vial using a SPEX 8000M Mixer/Mill and a ball to powder ratio of 10:1. The milled material was then charged into an in house made Sievert´s type apparatus and heated up to 450 °C under 1 bar of hydrogen pressure. The material was kept at 450 °C for about 6 h before cooling down to room temperature, as previously reported by some of the authors [30,31]. The purity of all materials was confirmed by X-ray powder diffraction and its analysis.

Muonic X-ray Emission spectroscopy was performed on the Port 4 (CHRONUS) of the RIKEN-RAL branch of the ISIS spallation source. Throughout the whole experiment, the beamline was tuned to extract surface muons with a momentum of 27 MeV/c and the beam was collimated with a lead shield having a 20 mm-diameter circular opening, resulting in a comparable beam-size.

A quantity of at least 250 mg of each sample was put inside titanium holders (24 mm-diameter) and sealed with 25 µm-thick titanium window suitable for the penetration of muons. All the samples were individually aligned in front of the beam and measured at room temperature for a collection time between 5 and 24 h per sample, depending on its density, affecting the muon stopping rate, and concentration of the ion of interest. X-rays were collected with four ORTEC Coaxial, GMX-Series Germanium detectors aligned at 30° with respect to the plane of the sample and disposed according to the setup of the RIKEN-RAL endstation reported elsewhere by some of the authors [10].

The detectors were calibrated prior to the experiments by measuring the gamma-ray emission from reference isotopes. Within the same calibration procedure, the electronic amplifiers of the detectors were tuned with respect to the intensity ratio of the reference sources, thus accounting for the differences in efficiency across the large investigated energy range [10]. Two electron counters were used together with the detectors to account for high-energy, scattered electrons and subtract this spurious component from the spectra. A functional background, accounting for the low-energy profile of the photoelectric absorption and the inelastic Compton scattering, was subtracted from all the raw spectra, and the intensity of X-ray peaks was estimated by fitting with Gaussian profiles the peaks due to muonic transitions. Such choice for the peak profile is justified because of the detector-limited experimental energy resolution. The emission lines identified in the text are labeled according to the Siegbahn notation. For the analysis of the emission from calcium, the Ca Kβ emission line overlaps with the Mα1 line from the lead of the beam collimator. We subtracted this contribution by fitting the intensity of the Pb Nα1 (437.1 keV) and Mα1 in a dedicated background scan and scaling the intensity of Ca Kβ peaks with respect to the Pb Nα1 emission fitted in the corresponding scan.

## 3. Results and Discussion

**Figure 2:**
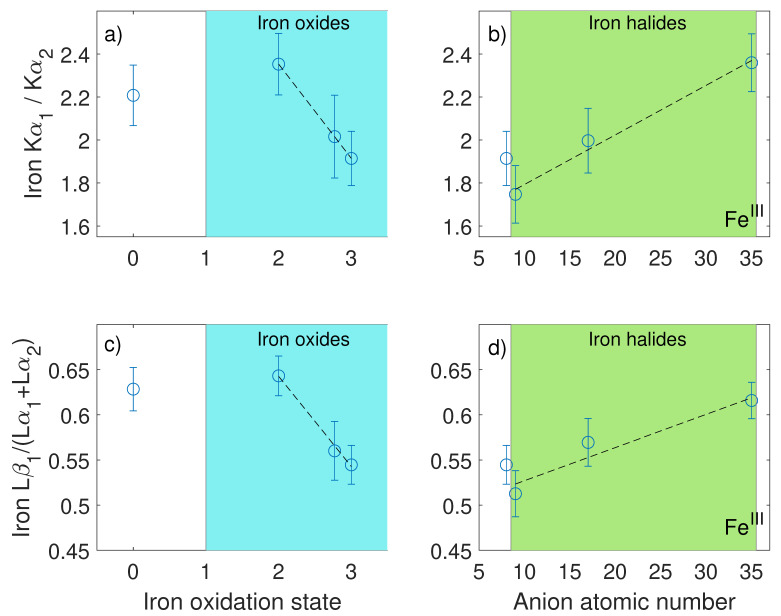
Intensity ratio of the Kα**_2_** (1253.7 keV) and Kα**_1_** (1257.9 keV) emission lines in iron oxides (panel **a**) and iron halides (panel **b**). Intensity ratio of the Lα**_2_** (265.7 keV) and Lα**_1_** (269.4 keV) in iron oxides (panel **c**) and iron halides (panel **d**). Dashed lines are guides for the eyes.

Selected spectra of iron compounds are shown in Figure 1b,c, while Figure 2 presents the results of a systematic investigation of the emission lines from iron contained in pure oxides and halides. Within the first family of compounds, we measured anhydrous FeO, Fe_3_O_4_ (magnetite) and Fe_2_O_3_ (hematite); the second group includes FeF_3_, FeCl_3_, and FeBr_3_. The results from pure Fe metal are shown for reference in Figure 1a,c; equivalently, the results for Fe_2_O_3_ are added for comparison in Figure 1b,d. Iron atoms show a clearly observable muonic X-ray emission at the energies of 1253.7 keV and 1257.9 keV, corresponding to the Kα**_1_** and Kα**_2_** transitions, respectively. The photons from the L transitions are observable at the lower characteristic energies of 265.7 keV (Lα**_1_** + Lα**_2_**) and 269.4 keV (Lβ**_1_**).

**Figure 3:**
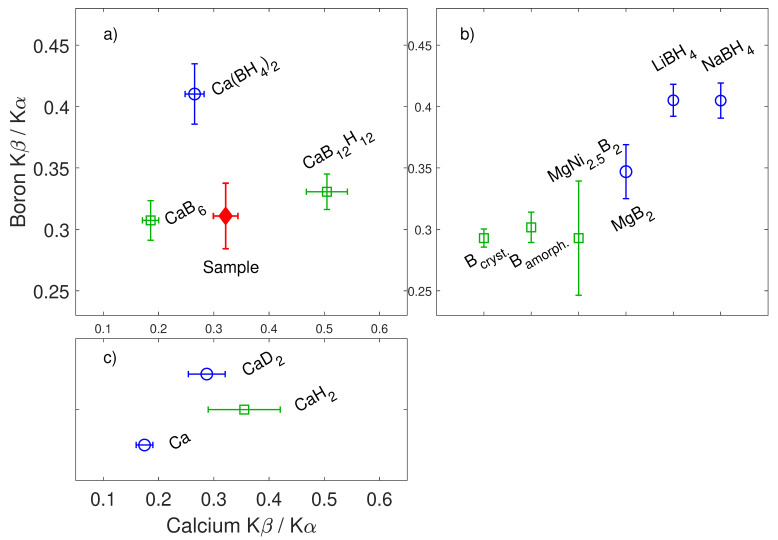
Intensity ratio of the Kα and Kβ emission lines in boron- (**a**,**b**) and calcium- (**a**,**c**) containing compounds. Panels a and b have a common vertical axis, and panels a and c share the horizontal one for comparison. The sample of reacted Ca(BH_4_)_2_-Mg_2_NiH_4_ is marked with a red diamond; the known reaction products are shown as green squares, while other reference compounds of interest for the discussion are labeled with blue circles.

In Figure 2a,b, the ratios of the Kα**_1_** and Kα**_2_** emission lines are shown, corresponding to the transitions from the muonic 2p**_3/2_** or 2p**_1/2_** states to the 1s**_1/2_** state, respectively. Similarly, Figure 2c,d contains the ratio of the Lα**_1_** and Lα**_2_** lines. The energy resolution in such hard X-ray range prevents the analysis of the separated Lα and Lβ lines, resulting from allowed transitions among M**_1-5_** to L**_1-3_** levels. This work is presented according to the more general Siegbahn notation, but the reader should be aware that Lβ lines include the contributions from near-degenerate M**_4_**-L**_2_**, M**_3_**-L**_1_**, and M**_2_**-L**_1_** transitions. The Kα**_1_**/Kα**_2_** ratio shows a systematic decrease in value corresponding to an increase in the oxidation state of Fe. The data collected in the iron halides are characterized as well by a similar trend, and the ratios increase when the atomic number of the anion increases. After observing that the emission of muonic X-rays shows systematic behavior with respect to the local chemistry of elements as in XANES spectroscopy [2], the application of this method for the chemical identification of energy-relevant materials and compounds of light elements is here tested. The properties of the characteristic emission lines of boron and calcium are explored in a sample of Ca(BH_4_)_2_-Mg_2_NiH_4_ and in reference materials of interest for its dehydrogenation reaction [30]. The study of this challenging chemical reaction has paramount importance, because the couple delivers the first observed condition where boron is accepted by the reacting partner Mg_2_NiH_4_ and reversibly returned to the hydride Ca(BH_4_)_2_ upon changing applied temperature and hydrogen pressure conditions [30]. This determines a model approach for the application of targeted complex metal hydrides in solid-state H_2_ storage solutions [31]. The test of µ-XES with such a complex reaction constitutes a proof of concept for its application as a qualitative analytical tool complementary to XRD and XAS for the study of light elements and non-crystalline materials. Due to the relatively small spin orbit coupling at the boron and calcium 2p_1/2_ and 2p_3/2_ states, it is not possible to resolve the different components of the Kα lines. Therefore, the ratio Kβ/Kα has been considered, and Figure 3 shows the comparison of such ratio for relevant calcium and boron containing compounds. The authors’ decision is also motivated by the trend observed at the K and L emission lines. In both iron oxides and halides, the measured variation is remarkably larger at the K edge than at the L edges, hinting towards a stronger dependence of the former on compound-specific features.

The Kβ/Kα ratio of the known dehydrogenation products is marked with green squares, while the reagents and other references are marked with blue circles. The value of the Ca Kβ/Kα ratio in the dehydrogenated sample, marked with a red diamond, is clearly compatible with the presence of CaH_2_, while the B Kβ/Kα ratio suggests a series of boron containing materials, namely, MgNi_2.5_B_2_, elemental B in both crystalline and amorphous form, and possibly MgB_2_. During the decomposition reaction of Ca(BH_4_)_2_, calcium atoms are involved in the formation of CaH_2_ and minor fractions of CaB_6_ and CaB_12_H_12_, while MgNi_2.5_B_2_ is formed as a consequence of the capture of boron by the reactive partner Mg_2_NiH_4_. The transfer of boron, however, is not complete, and a stoichiometric fraction of boron remains trapped in amorphous boron and in minor amounts of CaB_6_ and CaB_12_H_12_. These compounds are elusive to diffraction, but some of the authors previously showed their presence by using the local probes of nuclear magnetic resonance and XAS, and demonstrated that CaB_6_ and CaB_12_H_12_ involve less than 4% of available calcium [30]. They are therefore expected to be weakly contributing to the Ca Kβ/Kα ratio recorded in the herein-presented sample.

**Figure 4:**
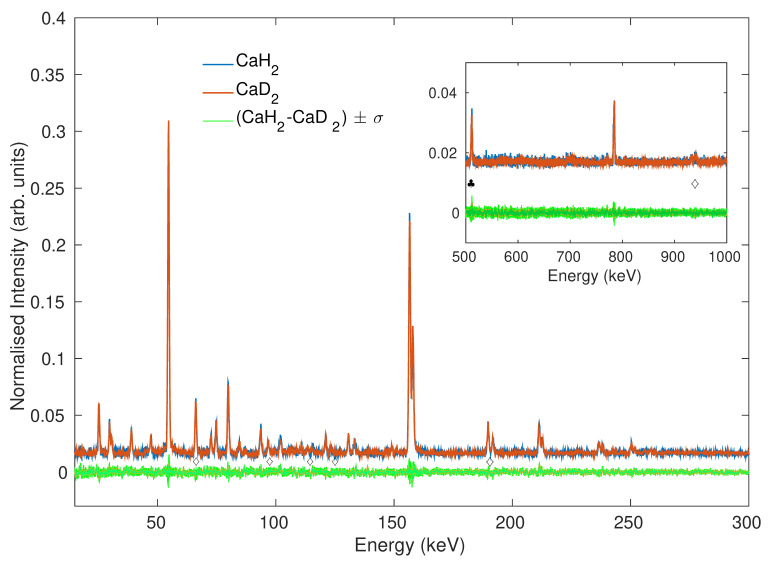
High statistic muonic X-ray Emission Spectroscopy (µ-XES) spectra of CaH_2_ (blue line) and CaD_2_ (red line) and difference (green line). A functional form of the background, accounting for the low-energy profile of photoelectric absorption and Compton scattering, was fitted and subtracted from both the spectra, which were then normalized over the entire scattered intensity. The Inset shows the zoom of the high-energy portion of the spectra. The diamond markers identify the peaks belonging to titanium sample holders, while the club marker (in inset) shows the positron-annihilation emission line at the energy of 511 keV.

Remarkably, µ-XES clearly suggests the presence of elemental boron, as well as all other products. The study of the Kβ/Kα ratio thus provides a clear qualitative analysis of the reaction capable of suggesting the presence of the correct chemical species. The origin of the sensitivity of the muonic X-rays to the chemical structure is now discussed. It is worth pointing out that the emission of characteristic X-ray lines following the implantation of negative muons has been generally described [32]. A few previous works by Naumann [25], Knight [24] and Von Egidy [33] have highlighted the existence of differences in the Balmer and Lyman series of ions belonging to different compounds, but this previous research focused on presenting and explaining the details of the element- and compound-dependent capture ratio for negative muons. In its oxides, iron atoms bear different oxidation states corresponding to the occupation of different coordination shells. In FeO, Fe**^II^** determines a tetrahedrally coordinated site surrounded by four oxygen atoms whereas Fe**^III^** in Fe_2_O_3_ coordinates six O_2_^−^ ions disposed in octahedral coordination. In Fe_3_O_4_ (magnetite), Fe**^II^** and Fe**^III^** coexist, leading to an intermediate formal oxidation state. This suggests an explanation involving either the different oxidation state or the spatial distribution of neighboring atoms. In the series of halides, Fe**^III^** occupies the center of octahedra at average distances from the anions of 1.923(8)Å in FeF**_3_**, of 2.378(5)Å in FeCl_3_ and of 2.136(6)Å in FeBr_3_. With respect to the first coordination shell, the three compounds are comparable. The different bonding distances arise from many factors, i.e., from the different electronegativity and ionic radii of the anions, but the main local difference on the site of Fe is the electron density provided by the first-neighbor anions. The results in this family of materials thus foster the picture of an effect of the first coordination shell on µ-XES spectra. This consideration can also explain the differences at the B and Ca Kβ/Kα ratios shown in Figure 3 for several compounds. Interestingly, a comparable B Kβ/Kα ratio is observable for the complex borohydrides LiBH_4_, NaBH**_4_**, and Ca(BH_4_)_2_. These three compounds have indeed different crystallographic structures (Pnma, Fm-3m, and either F2dd or P-4, respectively), but all show boron ions occupying identical BH_4_^−^ units. The local electronic structure at the site of boron atoms is therefore comparable, thus supporting the thesis of a B Kβ/Kα ratio comparable for the three compounds. This expected result is indeed clearly observable in the Figure 3. In order to explore if the change in the distribution of the electron-density is the right explanation for the sensitivity of µ-XES to the chemistry of elements, spectra collected from the same element in allotropes or isostructural compounds resulting from isotopic substitution, namely, amorphous and crystalline boron, as well as CaH_2_ and CaD_2_, have been considered. The Kβ/Kα ratio in boron-containing allotropes is equivalent. Nevertheless, this result is not conclusive because of the structural differences between crystalline and amorphous boron affecting the electron density. The Ca Kβ/Kα ratio is also equivalent in CaH_2_ and CaD_2_. High-statistic spectra for these two compounds are shown in Figure 4.

When the emission lines from the two isostructural CaH**_2_** and CaD**_2_** compounds are considered, no noticeable differences can be found in the Ca Mα, Lα, and Kα emission lines. This result is clearly supporting the suggested dependence of µ-XES on the electron-density, and thus on the electronic-structure of the material, which is equivalent upon isotopic substitution. Nevertheless, this analysis should also consider and address the nuclear effects related to the isotopic substitution in the first coordination shell of calcium and thus describe the contribution of the transfer of negative muons from H and D atoms to Ca [34]. The transfer of µ-has been mainly investigated in isolated elements such as noble gases [35,36,37]. It is established that µ- occupying the 1s state of hydrogenoid atoms can transfer to other atoms and the transferred µ- preferentially populates low angular momentum states [38]. It is not possible to exclude that such event could artificially alter the probability of the muonic transitions and thus the Kα and Kβ intensities. Such suggestion is also in agreement and supported by the previous observation of the dependence of the hydrogen Kα, Kβ, and Kγ intensities on the density of the gas [39]. However, Figure 4 shows that the muon transfer does not influence our measurements to a relevant extent. The difference of the scattered intensity in CaH_2_ and CaD_2_ does not exceed 3% and is comparable with the error distribution in our measurements. While the effect of the muonic-transfer on the emitted spectra in all H-containing materials cannot be excluded, the absence of differences in CaH_2_ and CaD_2_ help argue that the extent of this contribution should be marginal. Furthermore, muon transfer is less likely to affect heavier elements, as suggested by the systematic decrease in the muon transfer when comparing H to D [40]. It is therefore reasonable to conclude that the materials-dependent modulation of intensity of emitted X-rays, which we highlighted in µ-XES spectra, is mainly affected by the local electronic-structure of the material. It is worth discussing some of the existing literature, in order to suggest a description on how this effect takes place. When considering µ-XES, it is easy to suggest an explanation based on its analogy with the more established XES technique. The semicore and deeper core transitions involved in XES are often reasonably described by atomic multiplet approaches due to the localization of the atomic-like initial and final states [41]. The situation is markedly less clear for the transitions corresponding to the emission of muonic X-rays. Previous calculations on light elements suggest that most of the electrons are stripped from the atom during muon-capturing processes because of the excessive electron-binding energy of the atom after capturing a µ^−^ [42]. However, this event of extreme ionisation should be characterized by an extremely short lifetime and followed by dynamics of recapture of electrons from the conduction band or electrons from other atoms. Previous calculations on iron metal [43] have shown that the rate for the refilling of a vacancy in the electronic K shell is 1.6 × 10^15^ s^−1^, while the corresponding rate in the L shell is approximately 7 × 10^14^ s^−1^. Since the muonic de-excitation cascade to the lower levels occurs on a timescale of the order of 10^−14^ s [44], it is clearly possible to notice that the muon transitions should happen in presence of electrons occupying the 1s_1/2_ electronic shells and potentially on the same timescale of the recombination at the L states. The observation of µXES sensitivity to the chemical state, as shown for the K lines in Figure 2, Figure 3 and Figure 4, implies that the local environment affects the expected statistical population of 2p_3/2_ and 2p_1/2_ states, namely, 2:1. While one would point to a transition between the states 2p_3/2_ and 2p_1/2_, the lifetime of muonic states [43] also gives us the possibility to exclude these transitions involving the magnetic spin as considerably slower than those with selection rules Δn = 1 [43]. As an explanation for these results, an effect that modulates the population of muonic levels during the muonic cascade should be considered. Vogel thoroughly studied the effect of the electronic screening on the muonic binding energy and thus on muonic X-ray emission in a few atoms, showing that approximately 80–85% of the observable screening effect comes from the 1s_1/2_ electrons [45]. Such an effect influences the muonic states by establishing a connection between their probability of population and the average number of atomic 1s electrons present when µ^−^ has reached the corresponding n or l state. Hence, indirectly, a variable population of muonic states connected to a material-dependent screening suggests an explanation for the molecular effects that are observable at both K and L emission lines [45]. Despite the fact that the effect of screening provides a viable qualitative explanation to the herein-presented data, the influence of the distribution of angular momentum should also be considered, as suggested by Naumann and coauthors, to explain the differences in the muon cascade in Fe^III^ compounds [25]. Such effect is reported to be independent on the l quantum number. In order to be able to confirm its presence, we should map an X-ray emission corresponding to the transitions with selection rules Δn = 0 and Δl = 1. Such transitions have not yet been reported in muonic X-rays, probably due to their unfavorable probability. While the contributions of the electronic screening and of the momentum distribution on the muon cascade cannot be separated, it can be concluded that the spatial distribution of electrons is the main motivation for the sensitivity of the muonic emission lines to the electronic structure of materials.

## 4. Conclusions

This study shows that the intensity of the muonic Kα and Kβ emission lines are reminiscent of structural and molecular features and therefore capable of identifying materials with different oxidation states; different structures; and, in general, with different local chemistries affecting the electronic-structure. This method is validated by showing its application to the study of energy-storage materials, namely, by exploring the products of the dehydrogenation reaction of the mixed hydride system Ca(BH_4_)_2_-Mg_2_NiH_4_, thus providing a proof of the possibility to identify materials based on light elements and amorphous products. This work demonstrates, for the first time, that µ-XES can be successfully used as a local probe for the chemistry of materials beyond its established use for the elemental analysis, and suggests a wide range of applications for this technique thanks to the compatibility of muon spectroscopy with in-situ, in operando, and high-pressure sample environments [46,47].
